# EMDR for Syrian refugees with posttraumatic stress disorder symptoms: results of a pilot randomized controlled trial

**DOI:** 10.3402/ejpt.v6.27414

**Published:** 2015-05-18

**Authors:** Ceren Acarturk, Emre Konuk, Mustafa Cetinkaya, Ibrahim Senay, Marit Sijbrandij, Pim Cuijpers, Tamer Aker

**Affiliations:** 1 Department of Psychology, Istanbul Sehir University, Istanbul, Turkey; 2 Institute for Behavioral Studies, Istanbul, Turkey; 3 Kilis Refugee Camp, Kilis, Turkey; 4 Department of Clinical Psychology, VU University Amsterdam, Amsterdam, The Netherlands; 5 School of Medicine, Department of Psychiatry, Kocaeli University, Kocaeli, Turkey

**Keywords:** Refugees, posttraumatic stress disorder, depression, randomized controlled trial, psychotherapy

## Abstract

**Background:**

The most common mental health problems among refugees are depression and posttraumatic stress disorder (PTSD). Eye movement desensitization and reprocessing (EMDR) is an effective treatment for PTSD. However, no previous randomized controlled trial (RCT) has been published on treating PTSD symptoms in a refugee camp population.

**Objective:**

Examining the effect of EMDR to reduce the PTSD and depression symptoms compared to a wait-list condition among Syrian refugees.

**Method:**

Twenty-nine adult participants with PTSD symptoms were randomly allocated to either EMDR sessions (*n=*15) or wait-list control (*n=*14). The main outcome measures were Impact of Event Scale-Revised (IES-R) and Beck Depression Inventory (BDI-II) at posttreatment and 4-week follow-up.

**Results:**

Analysis of covariance showed that the EMDR group had significantly lower trauma scores at posttreatment as compared with the wait-list group *(d=*1.78, 95% CI: 0.92–2.64). The EMDR group also had a lower depression score after treatment as compared with the wait-list group (*d=*1.14, 95% CI: 0.35–1.92).

**Conclusion:**

The pilot RCT indicated that EMDR may be effective in reducing PTSD and depression symptoms among Syrian refugees located in a camp. Larger RCTs to verify the (cost-) effectiveness of EMDR in similar populations are needed.

The United Nations High Commission for Refugees (UNHCR) reported that in 2012 there were 15.4 million refugees in the world (UNHCR, 2013a). Including internally displaced people, asylum seekers, and refugees, there are 47 million people forcibly displaced worldwide, which is the highest number since 1994 (UNHCR, 2013a). In the last 2 years, the conflict in Syria has forced many people to flee their home country to find a safer place. According to UNHCR reports, in 2013 there were 2,846,186 displaced Syrian people worldwide (UNHCR, 2013a).

Refugees are forced to leave their home countries because of war, disease, starvation, or ethnic cleansing (UNHCR, 2004). They are likely to have been exposed to a number of traumatic events such as the threat of death; torture; starving or serious injury; and the injury, death, or disappearance of family members. The difficulties of living in a conflict area, problems during the journey, the experience of torture (Mollica et al., 1998), separation from family (Rousseau, Mekki-Berrada, & Moreau, 2001), and having a prior trauma (Trautman et al., 2002) are all found to be related to mental health problems among refugees. One of the priorities in emergencies is to protect and improve people's mental health and psychosocial well-being (Inter-Agency Standing Committee [IASC], 2007). Recently, the UNHCR recommended that mental health services for refugees be increased and strengthened (UNHCR, 2013a).

Besides the past traumatic events, refugees may also have worries about their future. A recent study of refugee psychiatric outpatients in Norway indicated that postmigration stressors such as unemployment, poor social integration, and weak social network are related to mental health problems (Teodorescu et al., 2012).

When repeated and prolonged traumatization, as well as difficulties with living in exile, are combined with worries about the future, the risk for mental health problems such as depression, anxiety disorders, and posttraumatic stress disorder (PTSD) increases. De Jong, Scholte, Koeter, and Hart (2000) reported that 50% of the refugees in Rwandan and Burundese camps had serious mental health problems. A study of Cambodian refugees living in the Thailand–Cambodia border camp indicated that 55% had depression and 15% had PTSD (Mollica et al., 1993).

However, studies evaluating the efficacy of psychological treatments for PTSD carried out in refugee camps are very rare (Nickerson, Bryant, Silove, & Steel, 2011). Neuner et al. (2008) conducted two randomized controlled trials (RCTs) on the efficacy of narrative exposure therapy, psychoeducation, and supportive counseling in refugee camps in Uganda. They found that narrative exposure therapy was superior in reducing PTSD symptoms compared to the other interventions.

Eye movement desensitization and reprocessing (EMDR) is a psychological treatment for PTSD that involves a client recalling traumatic memories while simultaneously making horizontal eye movements or engaging in other bilateral stimulation, such as tapping (Shapiro, 2001). EMDR is an effective treatment for PTSD (Bisson et al., 2007; Bradley, Greene, Russ, Dutra, & Westen, 2005; World Health Organization, 2013), and its use is recommended in clinical guidelines (National Institute for Clinical Excellence [NICE], 2005; World Health Organization, 2013). However, no studies evaluating the efficacy of EMDR in refugee camp settings have been carried out yet. One pilot RCT on the use of EMDR as stabilization treatment for PTSD symptoms in refugees seeking asylum in the Netherlands revealed that EMDR was feasible and acceptable (Ter Heide, Mooren, Kleijn, De Jongh, & Kleber, 2011). Note that this pilot study had a very small size (*N*=20), limiting conclusions about EMDR's efficacy in this population. Moreover, the included refugees and asylum seekers were not located in camps, but were living in the Netherlands for an average of 10 years.

The war/conflict in Syria has so far displaced more than 1.5 million people (UNHCR, 2013b). Turkey is the third country of emigration after Jordan and Lebanon for Syrian refugees who are forced to flee. As of November 7, 2013, there are more than a half million Syrian refugees in Turkey and 513,157 had been registered as refugees or have registration appointments. More than half of them are in urban areas, whereas 202,379 are registered in 20 camps (UNHCR, 2013c). As refugees are thought to constitute a risk group for mental health problems, we aimed to provide a psychological intervention to those who need it. However, psychological treatment is not common or well-accepted in Syria. Moreover, resources to provide mental healthcare to those refugees with PTSD symptoms are not adequate.

Considering the limited human and monetary resources in refugee camps, we aimed to find a cost and time effective, short therapy. Therefore, we planned an RCT with Syrian refugees utilizing EMDR therapy (Shapiro, 2001) compared it to those in a wait-list group. Care was taken to ensure that the most relevant traumatic experiences encompassing the entire stay at the camp were addressed (Shapiro & Laub, 2008). This study is a pilot exploratory RCT to examine the feasibility, acceptability, and efficacy of EMDR therapy as a treatment of PTSD for this population.

## Method

### Ethics statement

The study was reviewed and approved by the Ethics Committee of Istanbul Sehir University (IRB Protocol 04/2013). The study was registered to Clinical Trials **(**ClinicalTrials.gov Identifier: NCT01847742). Participants provided their written informed consent to participate in this study

The consort checklist is available as supporting information (see Checklist_S1). This study is a single-blind, parallel-group, open-label RCT with two groups: the EMDR intervention and a wait-list control group (WL). The participants who were eligible for the study were approached and those who consented to participate were randomly assigned to either the EMDR or the control condition.

### Participants and procedure

The pilot RCT was conducted between April 2013 and July 2013 in Kilis Refugee Camp, which is located at the border between Turkey and Syria.

To construct the study population, 820 adult refugees aged 18 or over were randomly selected from a total of 14,000 refugees living in the camp. The selection was conducted by using a computer-generated random number list. Six hundred and eighty-eight (83.5%) of the selected refugees had scores on the Impact of Event Scale-Revised (IES-R) above the predetermined cutoff point (≥33; Creamer & Falilla, 2002) of probable PTSD. Of these 688 individuals, 45 participants were randomly selected by using a computer-generated random number list. First, 30 sets of four people (120 people in total) were randomly selected from the 685 people with PTSD symptoms. Next, the four randomly chosen people in each set were randomly ordered. The potential participant who was randomly ordered to be the first was approached first. If he or she refused to participate, the person randomly ordered to be the second was contacted, etc.

Inclusion criteria were the following: aged 18 and older, and having PTSD symptoms (IES-R score ≥33). Exclusion criteria were: having mental retardation, being pregnant, and using psychiatric medication.

**Fig. 1:**
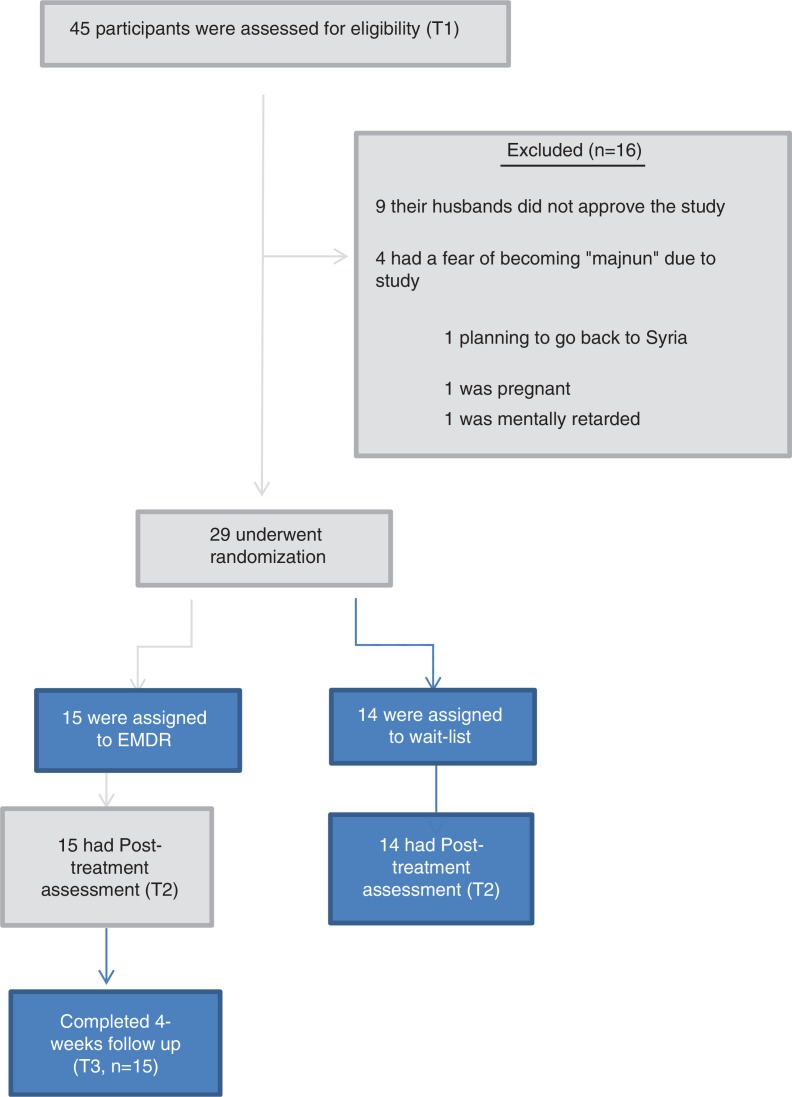
Consort diagram showing the flow of participants through each group. Fifteen participants were in the EMDR condition and 14 participants were assigned to wait-list control condition. The control group did not receive any pharmacological or psychological treatment while the EMDR group received a maximum of seven sessions of treatment (mean=4, 13).

We approached 45 people who were not actively seeking treatment. In total, 16 people refused to participate. Nine women refused to participate because their husbands did not allow them; individuals said that they heard that any psychotherapy would make them “majnun” (insane in Arabic), and one individual decided to go back to Syria. One person was excluded because of intellectual disabilities and one due to pregnancy. We randomly assigned the remaining 29 participants to groups (the EMDR intervention *N*=15 and the WL control condition *N=*14). The flow chart in Fig. 1 presents the progress of participants through the trial. There was no dropout from treatment in the EMDR group, or during completion of the assessments.

Informed consent was collected from all participants prior to the pretreatment assessment.

Assessments were scheduled at pretreatment (IES-R and Beck Depression Inventory (BDI)) and at posttreatment at 7 weeks following the pretreatment assessment (IES-R and BDI). In the EMDR condition only, a follow-up assessment (IES-R and BDI) was scheduled at 11 weeks after the pretreatment assessment. Participants in the control condition received only posttreatment assessment due to logistic (or technical) problems. Refugees have a high mobility rate, so in longer follow-up periods we anticipated high dropout rates. Based on our concerns and other studies with comparable follow-up times, we planned 4-week follow-up periods.

All questionnaires were self-report instruments, but for those who needed help, a research assistant who was blind to the treatment conditions administered the scales verbally.

### Randomization and blinding

For the allocation of participants to different treatment groups, a computer-generated random number list was used. Participants were randomly assigned on a 1:1 basis to the EMDR or wait-list group. The participants and the therapists were aware of the allocated arm, but the outcome assessors were kept blind to the allocation.

## Intervention

### Eye movement desensitization and reprocessing therapy

In this study, a maximum of seven sessions (90 min per session) of EMDR were conducted. Each treatment consisted of eight phases. Phase 1 consists of history taking, case formulation, and treatment planning. In Phase 2 the client was prepared for EMDR therapy by explaining EMDR therapy. Participants also learned self-calming techniques in this stage. In Phase 3, a target trauma memory was selected to work on. For this study, the worst trauma memory was selected as the target and only traumatic memories from the beginning of the war until the present time were targeted. As part of the Phase 3, a negative cognition about self that was associated with the worst memory, a positive cognition about the self (what the client wants to believe about himself), the validity of the positive cognition, the feelings experienced about the memory, (score on subjective unit of disturbance about the memory), and where in the body the disturbance is felt were elicited from the client. These indices help the therapist and the client to monitor how the session progresses, while making the client fully focus on and activate the memory. Phase 4 is the desensitization phase. The therapist asks the patient to hold the target trauma memory in mind for 30 seconds. During that time, the patient follows the finger of the therapist that moves from left to right across the patient's visual field, or the therapist used tapping. The patient reports current sensations, cognitions, and emotions. Sets are repeated until the client reports minimal distress associated with the memory. Then, the installation of the positive cognition is conducted with the bilateral stimulation. After the body scan, in the closure phase, if the clients were using religion to relax, we told them to imagine a holy light coming from heaven. This was seen as desirable in their culture.

The EMDR treatments were delivered by five Turkish psychologists who were trained at the EMDR level I by the second author who is an EMDR Institute accredited trainer. The EMDR trainer provided face-to-face and online live (Skype) supervision weekly.

### Cultural sensitivity

In order to be culturally sensitive and to prevent drop out we adopted specific measures. First, all interviews were carried out in the local language, with the help of Syrian interpreters. Second, in order to decrease the possible prejudice against mental health service use, psychoeducation related to trauma, PTSD, and EMDR was provided to Syrian opinion leaders at the camp, such as imams, village head men, and some women who have strong social networks. Third, we scheduled sessions in the late afternoon because in Syria people prefer to stay up late in the evening and wake up later in the morning, due to high temperatures in the area.

Moreover, possibly related to perceived stigma, refugees preferred to hide the fact that they were receiving treatment. Our clinic was at the kindergarten building in the camp. The participants did not want to be labeled as “majnun” (insane), so they were bringing their children to the building to pretend that they were coming to the kindergarten. For that reason, someone in our team was taking care of the child while the mother had the session. Fifth, we tried to ensure a match between the sex of the therapist and the client. But if that was not possible, we matched the sex of the interpreter with the client.

### Treatment fidelity

None of the participants gave permission for the video- or audiotaping of the sessions. The reason reported for refusal was fear of the Syrian government. For that reason, the supervisor personally observed a minimum of one session with each therapist (with the permission of the participant). The supervisor checked during live and normal one-on-one and group supervision sessions whether the therapists were complying with the 8 Phase EMDR Standard Protocol (Shapiro, 2001). Treatment fidelity was supported by the supervisor, who attended at least one session of each therapist.

### Wait-list control

The wait-listed, control group did not receive any psychological or pharmacological treatment in the camp or outside the camp. They were informed that after the study they could receive psychological help from the research team.

### Measures

#### The Impact of Event Scale-Revised

The efficacy of the treatment was assessed by comparing the total IES-R scores for the EMDR group and the wait-list control group (Weiss & Marmar, 1997). The IES-R is a 22-item self-report instrument which rates the severity of PTSD symptoms. Participants rated each item on a five-point Likert scale from 0 (not at all) to 4 (extreme). IES-R total scores range between 0 and 88, with higher scores indicating higher levels of PTSD symptoms. There are three subscales of IES-R: re-experiencing/intrusion, avoidance/numbing, and hyperarousal. The validity of IES-R has been tested in different populations (Panahi et al., 2011). We used a cutoff score of ≥33 as indicating the presence of posttraumatic stress symptoms (Weiss & Marmar, 1997). The scale was translated into Arabic by two independent translators. After back translation, conflicts arising between the original translation and the back translation were discussed by a group of professionals (Zaghrout, 2013). Administration of the scale in a sample of native Arabic speakers (Zaghrout, 2013) yielded a Cronbach's alpha of *α=*0.93. The test–retest reliability calculated by administering the scale to the same sample on two occasions, 2 weeks apart, yielded a Pearson correlation coefficient of *r*=0.88 (Zaghrout, 2013).

#### Beck Depression Inventory-II

Depression symptoms were measured with the BDI-II which is a widely used self-report instrument with satisfactory psychometric properties. The Arabic version of the BDI-II was developed by Ghareeb (2000), which included Syrian participants as well as participants from 17 other Arabic groups (as cited in Bader, 2006). The BDI-II has 21 items and the total score varies between 0 and 63, with higher scores indicating more severe depression (Beck, Steer, & Brown, 1996). A score of 21 or higher indicates moderate depression, and a score lower than 10 is considered to indicate the absence of depression.

### Data analysis

Statistical analyses were performed with SPSS version 19.0. Baseline differences in demographic data and pretreatment measures were analyzed by using *t*-tests. Differences between the EMDR group and control group and pre- to posttreatment changes in outcome measures were analyzed using *t*-tests as well as univariate analyses of variance and covariance (i.e., ANOVAs and ANCOVAs). In the ANCOVA model, the pretreatment scores were included as a covariate. For the treatment condition, the long term treatment effect was analyzed using ANOVAs and *t*-tests as planned contrasts. Between- and within-group effect sizes were calculated in terms of Cohen's *d* to allow for a comparison with previous reports that often relied on *d* to calculate effect sizes. Cohen's d was calculated as the difference of means divided by the pooled standard deviation. All analyses were carried out with *p*<0.05 indicating statistical significance.

## Results

### Baseline data

The majority of participants were female (*n*=22, 75.86%). The sociodemographic data did not differ significantly between two groups (Table 1). The mean number of EMDR sessions was 4.13 (SD=1.73, range=2–7).

**Table 1:** Sociodemographic and migration related characteristics of the sample

	EMDR	WL	
Sex			
Female	11	11	
Male	4	3	
Age range	19–63	27–60	
M (SD)	35.27 (13.21)	37.92 (9.06)	*t*(26)=0.61, *p*=0.55
Education (in years)	6.07 (4.29)	5.08 (4.19)	*t*(25)=0.61, *p*=0.55
Duration at the camp (in months)	14.44 (4.25)	14.43 (4.99)	*t*(21)=0.01, *p*=0.99

The Kolmogorov–Smirnov test for normality indicated that the distribution of the number of sessions did not deviate significantly from a normal distribution (*D*= 0.197, *p*=0.120). This shows that the number of sessions was not biased in a particular direction of being greater or smaller in number, but was the result of a random process.

Furthermore, when we split the EMDR group according to the number of sessions they attended based on their median number of sessions completed (those attending less than four sessions, *N*=6 versus those attending four and more sessions, *N*=9), the independent-Samples Mann–Whitney *U* test and the Wilcoxon W test indicated that the rank-ordering of the pretest IES scores was not distributed the same across the two groups (*U*=48, *W*=93, *p*=0.01). The pretest IES scores’ mean rank was higher (mean rank=10.33 vs. 4.5) for those who attended relatively more number of sessions. However, there was no difference between the groups in terms of pretest BDI scores (*U*=38, *W*=74, *p*=0.08). The pretest BDI scores had a mean rank order of 5.17 versus 9.25 for the low and the high attendance groups, respectively.

In terms of demographics, the independent-Samples Mann–Whitney *U* test and the Wilcoxon W test indicated that the high and the low attendance groups did not differ in terms of age (*U*=18.5, *W*=63.5, *p*=0.33). The high and the low attendance group also did not differ in terms of their education level [χ(4)=5.489, *p*=0.24], the number of people they were living with [χ(3)=0.929, *p*=0.82], the marital status [χ(2)=2.046, *p*=0.36], and their sex [χ(1)=2.784, *p*=0.10].

### Posttraumatic stress symptoms and depressive symptoms

Table 2 reports the estimated means and standard deviations of IES-R and BDI-II on each assessment time for both groups.

**Table 2:** Means and standard deviations for IES-R and BDI-II scores

	EMDR (*N*=15)			WL (*N*=14)	
	
	T1	T2	T3	T1	T2
	Mean (SD)	Mean (SD)	Mean (SD)	Mean (SD)	Mean (SD)
IES-R	64.80 (12.08)	22.87 (20.27)	18.93 (20.31)	56.93 (7.15)	54.21 (16.26)
BDI	22.69 (7.77)	10.15 (9.60)	–	20.64 (8.71)	20.79 (7.92)

T1: pretreatment; T2: posttreatment, T3: 4 weeks follow-up.

**Table 3:** Statistical comparisons across time and groups

	Time		Time×Group		Posttest (EMDR vs. wait-list)		Pretest/Posttest (EMDR)		Posttest/Follow-up (EMDR)	
				
Measures	df	*F*-value	df	*F*-value	df	*F*- or *t*-value	df	*t*-value	df	*t*-value
_BDI_	1, 25	7.023*	1, 25	7.35*	25	3.15**	12	4.248***	–	–
_IES_			–	–	1, 26	24.166***	14	8.243***	14	1.33

* *p*<0.05

** *p*<0.01

*** *p*<0.001.

### PTSD symptoms

At pretreatment, IES-R scores were significantly higher in the EMDR group than in the control group (EMDR group: *M=*64.80, SD*=*12.08 vs. wait-list group: *M=*56.93, SD=7.15), *t* (27)=2.115, *p=*0.044, *d=*0.76, 95% CI (0.01, 1.52). Given the difference between the groups’ trauma scores at the baseline, we conducted an ANCOVA on the IES-R scores at the immediate posttest with the group (EMDR vs. wait-list) as the independent variable, and baseline trauma scores as a covariate. After correcting for the difference between the groups at baseline, as shown in Table 3, the EMDR group had significantly lower IES-R scores at posttreatment as compared with the wait-list group (*M=*22.87, SD*=*20.27 vs. *M=*54.21, SD*=*16.26), *F*(1, 26)=24.166, *p*<0.001, *d=*1.78, 95% CI (0.92, 2.64).

**Fig. 2:**
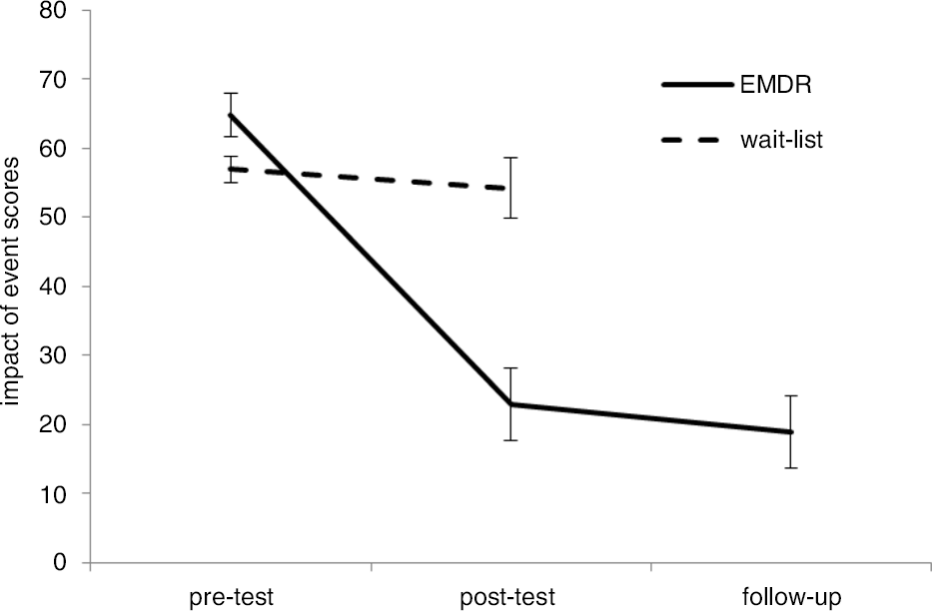
Change in the EMDR and the wait-listed groups’ Impact of Event Scores with Standard error bars across time.

We also compared the change in the trauma scores of the EMDR group across the baseline, the posttest, and the 1-month follow-up in a repeated measurement ANOVA. The ANOVA results indicated a significant effect of time, *F* (2, 28)=62.78, *p*<0.001, *d=*1.93, 95% CI (1.07, 2.8). As shown in Fig. 2, the EMDR group's trauma scores significantly decreased between the baseline and the immediate posttest (*M=*22.87, SD*=*20.27 vs. *M=*64.80, SD=12.08), *t* (14)=8.243, *p*<0.001, *d=*2.08, 95% CI (1.16, 2.99). Between the immediate posttest and the follow-up, the EMDR group's trauma scores did not change (*M=*18.93, SD*=*20.31 vs. *M=*22.87, SD*=*20.27), *t* (14)=1.330, *p*>0.1. In the posttest, 5 patients out of 15 in the EMDR condition had IES-R score of ≥33, and at the posttest, it was 3 out of 15. The wait-list group's trauma scores did not significantly change across the pre- and the immediate posttest, *t* (13)=0.611, *p*>0.10.

### Depression

There was no difference at the baseline between the groups’ depression scores (EMDR group, *M=*21.50, SD*=*8.7 vs. wait-list group, *M=*20.64, SD*=*8.71), *t* (26)=0.260, *p*>0.1. A 2×2 ANOVA with group (EMDR vs. wait-list) as between-participants factor, and time (pretest vs. posttest) as a within-participants factor, indicated a significant effect of time, *F*(1, 25)=7.023, *p=*0.014, *d=*0.49, 95% CI (0.05, 1.04), but no effect of the group in general, *F*(1, 25)=3.47, *p*>0.05. There was a significant time-by-group interaction *F* (1, 25)=7.35, *p=*0.012, *d=*0.51, 95% CI (0.04, 1.05).

**Fig. 3:**
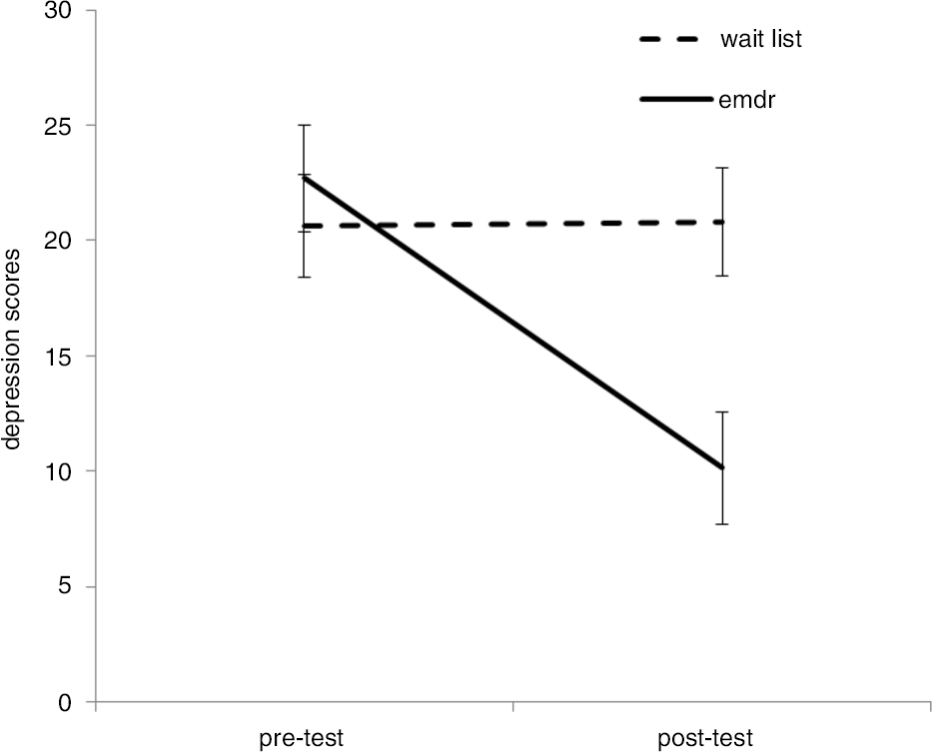
EMDR and wait-listed groups’ depression scores with standard error bars as a function of time.

As shown in Fig. 3, the planned contrasts indicated that after treatment, the EMDR group had lower depression scores (*M=*10.15, SD*=*9.60 vs. *M=*20.79, SD*=*7.92) than the wait-list group, *t* (25)=3.15, *p=*0.004, *d=*1.14, 95% CI (0.35, 1.92).

## Discussion

### Main results

Our results indicate that EMDR is efficacious in reducing symptoms of PTSD and depression among Syrian refugees living in a refugee camp. To our knowledge, no similar report has been published in the literature about the psychological treatment of refugees still located in a refugee camp. Moreover, our study suggested improvements in PTSD symptoms were maintained at 11-week follow-up. Note, however, that we do not have follow-up data for the wait-list control participants.

### Comparison with prior work

The results of our study are in line with previous studies indicating that EMDR is effective in reducing PTSD symptoms among adults (Van der Kolk et al., 2007). In the treatment of PTSD, EMDR and trauma-focused CBT were shown to be efficacious treatments (Bisson et al., 2007). Both EMDR and CBT led to higher reductions in PTSD symptoms than non-trauma-focused treatments such as psychodynamic therapy and supportive counseling. Recent international practice guidelines advocate the use of EMDR as a treatment of choice for PTSD (NICE, 2005; World Health Organization, 2013). Our study added to the evidence base, in that it showed that EMDR may also be beneficial for vulnerable non-western populations under high levels of current stress, such as Syrian refugees who are located in a refugee camp.

### Limitations

Besides the strengths of the current study, such as the population studies, and the fact that we used randomization to assign patients to groups, there are a number of limitations. One limitation is the lack of a formal diagnosis of PTSD in the research population. We included refugees with PTSD symptoms assessed on the basis of a self-report instrument. Due to practical and logistic difficulties, we could only conduct the study with a small number of participants. Also, absence of the 11-week follow-up assessment in the wait-list control group constitutes another limitation of the study. Moreover, treatment fidelity was not formally evaluated. Finally, although EMDR training consists of two levels (Level I and II), the therapists in the current study had received “Level I” training only. However, the supervisor who was an EMDR trainer watched at least one session of each therapist through Skype and provided individual feedbacks besides the group supervisions.

### Clinical and research implications

The present study reports promising results for EMDR as feasible, acceptable, and effective intervention in reducing PTSD and depression symptoms among refugees in a camp setting. However, to conduct an intervention in a refugee camp at the border was not easy for many reasons. As stated, some refugees were afraid of becoming insane as a result of psychological treatment. This could be partially explained by a low familiarity with mental help in Syria. A previous study about the mental health service use in Arab countries indeed indicated that in Syria there were fewer than 0.5 psychiatrists and no psychologists per 100,000 population in 2007 (IASC, 2007). Last, it is difficult to generalize our results to treatment-seeking populations, because our sample was not a treatment-seeking population. Despite these challenges, future studies with larger samples may evaluate the (cost-) effectiveness of EMDR and other psychosocial interventions for individuals located in refugee camps.

## Conclusion

Refugees have higher risk for mental health problems not only compared to host populations but also compared to other migrant groups (Bhugra et al., 2011). Moreover, given the increase in the number of refugees worldwide, it is important to conduct effective interventions both to reduce individual suffering and to prevent future conflicts in the communities.
